# Mapping analyses to estimate EQ-5D utilities and responses based on Oxford Knee Score

**DOI:** 10.1007/s11136-012-0189-4

**Published:** 2012-05-04

**Authors:** Helen Dakin, Alastair Gray, David Murray

**Affiliations:** 1 Department of Public Health, Health Economics Research Centre, University of Oxford, Old Road Campus, Headington, Oxford, UK; 2 Nuffield Department of Orthopaedic Surgery, University of Oxford, Oxford, UK

**Keywords:** Mapping, Cross-walking, Health-related quality of life, Joint replacement, Health state preference values

## Abstract

**Purpose:**

The Oxford Knee Score (OKS) is a validated 12-item measure of knee replacement outcomes. An algorithm to estimate EQ-5D utilities from OKS would facilitate cost-utility analysis on studies analyses using OKS but not generic health state preference measures. We estimate mapping (or cross-walking) models that predict EQ-5D utilities and/or responses based on OKS. We also compare different model specifications and assess whether different datasets yield different mapping algorithms.

**Methods:**

Models were estimated using data from the Knee Arthroplasty Trial and the UK Patient Reported Outcome Measures dataset, giving a combined estimation dataset of 134,269 questionnaires from 81,213 knee replacement patients and an internal validation dataset of 45,213 questionnaires from 27,397 patients. The best model was externally validated on registry data (10,002 observations from 4,505 patients) from the South West London Elective Orthopaedic Centre. Eight models of the relationship between OKS and EQ-5D were evaluated, including ordinary least squares, generalized linear models, two-part models, three-part models and response mapping.

**Results:**

A multinomial response mapping model using OKS responses to predict EQ-5D response levels had best prediction accuracy, with two-part and three-part models also performing well. In the external validation sample, this model had a mean squared error of 0.033 and a mean absolute error of 0.129. Relative model performance, coefficients and predictions differed slightly but significantly between the two estimation datasets.

**Conclusions:**

The resulting response mapping algorithm can be used to predict EQ-5D utilities and responses from OKS responses. Response mapping appears to perform particularly well in large datasets.

**Electronic supplementary material:**

The online version of this article (doi:10.1007/s11136-012-0189-4) contains supplementary material, which is available to authorized users.

## Introduction

Although condition-specific health-related quality-of-life (HRQoL) measures may be more sensitive and [1, 2] sufficient to assess efficacy, comparing incremental cost-effectiveness between conditions requires generic measures, such as the EQ-5D [3–5], that give health state preferences or utilities [1, 2]. Utilities are needed for calculation of quality-adjusted life-years (QALYs) and are scaled such that one equals perfect health, zero indicates death and negative values represent health states worse than death.

EQ-5D, the most widely used utility scale [6], includes five questions on mobility, self-care, pain, usual activities and anxiety/depression, each with three response levels [3–5]. Utility valuations for all 243 EQ-5D health states are based on time-trade-off valuations by 3,395 members of the UK general public [3, 4]. Alternative tariffs have been developed for other countries [7].

There is growing interest in algorithms that map one HRQoL measure onto another, thereby enabling estimation of QALYs from trials that include condition-specific measures but not utility instruments [8–10]. Most mapping studies use regression methods to directly predict utilities from responses or scores on condition-specific measures [8, 9]. However, response mapping models predicting patients’ responses to a multiattribute utility measure provide an alternative approach [9, 11–15]. Response mapping may provide better utility predictions as well as giving richer insights into the relationship between the two instruments, predicting the domains most affected by disease or treatment and calculating utilities for any tariff [11].

The Oxford Knee Score (OKS) is a widely used HRQoL measure that was developed and validated for the assessment of outcomes following knee replacement in comparative trials and cohort studies [16–18]. OKS is also increasingly used to assess eligibility for primary [19] or revision surgery [20], although it was not designed or validated for this purpose. It is also administered routinely to assess the performance of hospitals or surgeons in the UK and New Zealand Patient Reported Outcome Measures (PROMs) initiatives [21–24]. OKS includes 12 questions on knee symptoms and function, each with five levels. Scores on each question, which range from 4 (no problems) to 0 (severe problems), are summed without weighting to produce total scores ranging from 0 to 48 [18].

Since its development in 1998, OKS has been used in many large trials and cohort studies assessing the long-term durability of knee components that did not include utility measures [17]. A mapping algorithm predicting utilities from OKS would enable long-term data from these studies to inform cost-utility analyses.

This study estimates mapping models to predict utilities and/or responses to the three-level EQ-5D questionnaire based on responses and scores on the OKS. We also compare the performance of different mapping models and assess whether different datasets yield different mapping algorithms.

## Methods

### Data

#### Estimation datasets

Data from the Knee Arthroplasty Trial (KAT) and the UK PROMs initiative were combined to provide a large, diverse sample of knee replacement patients on which to develop a robust mapping algorithm and to test whether mapping models are sensitive to the dataset used. Following best practice [10] and to avoid over-fitting during model selection, 25 % of patients were allocated to the internal validation sample using computer-generated random numbers. All questionnaires from these patients were excluded from estimation models and were instead used to assess the prediction accuracy of each model and select the final model specification. The estimation sample and internal validation sample were then combined to estimate the final model, which was externally validated on a third dataset.

KAT comprised a randomized trial comparing different types of knee prosthesis, in which 2,352 patients underwent total knee replacement in the UK between 1999 and 2003. Patients completed OKS and EQ-5D pre-operatively, three and 12 months after knee replacement and annually thereafter for 8–11 years to date. All questionnaires received by 4 May 2011 that had complete responses to OKS and EQ-5D were included in mapping analyses, giving an estimation dataset of 12,961 questionnaires from 1,690 patients.

Within PROMs, all patients undergoing knee replacement in England are sent OKS and EQ-5D questionnaires pre-operatively and 6 months afterwards [22, 23]. We analyzed PROMs data on admissions for knee replacement up to 31 December 2010 that included 162,066 questionnaires with complete OKS and EQ-5D data from 106,320 patients. All questionnaires with complete data on EQ-5D and OKS were included in mapping analyses regardless of whether pre- and post-operative data were linked. This provided an estimation sample of 121,308 observations from 79,523 patients.

#### External validation dataset

The external validity of the best mapping algorithm was tested using a dataset from the Elective Orthopaedics Centre (EOC) that was not made available to the authors until after the final model was selected. This comprised a large observational cohort of patients undergoing hip or knee replacement at an NHS treatment centre serving four NHS trusts in South-West London from January 2004 onwards [25]. Patients completed EQ-5D and OKS pre-operatively and 6, 12 and/or 24 months afterwards. Although recruitment is ongoing, our analysis included only patients undergoing primary or revision knee replacement before 31 March 2009 to avoid overlap with PROMs. After excluding patients with incomplete data on OKS and/or EQ-5D, this external validation dataset included 10,002 observations from 4,505 patients.

### Statistical methods

#### Model estimation

We first estimated direct utility mapping models by regressing responses to individual OKS questions directly onto EQ-5D utility using four functional forms:

- Ordinary least squares (OLS) regression.
- Generalized linear models (GLM) with log link or gamma family predicting EQ-5D disutility (where disutility = 1 − utility), which allow for the skewed distribution of utility values and prevent prediction of utilities >1.
- Fractional logistic models, which constrain predictions to lie within the range determined by the EQ-5D tariff (1 to −0.594 [4]). These were implemented by using GLM with binomial family and logit link to predict utility_0–1_, where utility_0–1_ = (utility + 0.594)/1.594.

Two-part models were used to allow for the 9.6 % (17,184/179,482) of observations reporting perfect health (utility of one) on EQ-5D. For such models, the first part comprised a logistic regression model estimated on the entire estimation sample to predict which patients had perfect health, while the second part comprised an OLS model predicting EQ-5D utilities for those patients with utility <1.

We also developed and evaluated three-part models since 45.9 % (48,318/105,235) of pre-operative questionnaires indicated severe problems on ≥1 EQ-5D domain and therefore had substantially lower utility due to the N3 term in the EQ-5D tariff [4]. The first part of this model comprised multinomial logistic regression (mlogit) to predict whether patients had perfect health, severe problems on ≥1 EQ-5D domain or only mild–moderate problems. The second and third parts comprised OLS models to predict EQ-5D utility for the subset of patients with severe problems on ≥1 EQ-5D domain and for those with only mild–moderate problems, respectively.

We also used response mapping to predict the response level that patients selected for each of the five EQ-5D domains. These were estimated by fitting a separate mlogit or ordinal logistic regression (ologit) model for each EQ-5D domain, as described previously [11].

The explanatory variables for all models comprised 48 dummy variables indicating whether or not patients had a particular response level on each OKS question; response level 4 (no problems) comprised the comparison group. However, all models were also evaluated using two alternative sets of explanatory variables: 12 OKS question scores (rankings from 0 to 4); and total OKS (measured from 0 to 48 [18] based on unweighted summation of question scores). We also investigated whether adding sex into the best performing model improved prediction accuracy; to ensure that the mapping algorithm can be applied to all datasets, no other patient characteristics were added.

All models were estimated in Stata version 11 (StataCorp, College Station, TX). For all models, the cluster option within Stata was used to adjust standard errors to allow for clustering of observations within patients. Standard errors from two-part, three-part and response mapping models were also adjusted using seemingly unrelated regression to allow for correlations between EQ-5D domains [26].

#### Assessing model performance

Predicted EQ-5D utilities were estimated for each mapping model. Predictions from direct mapping models were estimated using the predict post-estimation command, with direct back-transformations applied to predictions from GLM and fractional logit models. For OLS models, any utilities predicted to be >1 were set to one. For two-part models, the expected utility for each patient was estimated as

1 $$ {\text{Utility = }}\Pr ({\text{Utility}} = 1) + (1 - \Pr ({\text{Utility}} = 1))U $$

where *U* equals the predicted utility conditional on imperfect health and Pr(Utility = 1) the predicted probability of having perfect health.

Similarly, for three-part models,

2 $$ {\text{Utility}} = \Pr ({\text{Utility}} = 1) + \Pr (N3)U_{N3} + (1 - \Pr ({\text{Utility}} = 1) - \Pr (N3))U_{{{\text{mild}} - {\text{moderate}}}} $$

where Pr(*N*3) indicates the probability of having severe problems on ≥1 domain, *U*_*N*3_ the predicted utility conditional on this and *U*_mild−moderate_ the predicted utility conditional on mild–moderate problems.

For response mapping models, the highest probability method (assuming that patients have the EQ-5D response level for which the predicted probability from multinomial/ordinal logistic regression is highest) has been shown to give biased predictions, and, in particular, underestimates the probability that patients will have severe problems [11, 14]. Instead, we generated predictions from response mapping models using the expected value method [14]. This is equivalent to the Monte Carlo method [11] given a large number of repeated Monte Carlo draws [14].

Models were selected based on the mean squared error (MSE) in the combined internal validation sample, where MSE equals the mean of squared differences between observed and predicted EQ-5D utility. Mean absolute error (MAE, the mean of absolute differences between observed and predicted EQ-5D utility) was also calculated.

Two further analyses assessed whether different datasets produced significantly different mapping models. Firstly, mapping models were estimated on the combined estimation dataset with a full set of interaction terms capturing the effect of data coming from KAT rather than PROMs on the coefficients for each OKS response. Secondly, each mapping model was re-estimated separately using each dataset to assess whether the model giving best predictions differed between datasets.

## Results

### Exploratory data analysis

**Fig. 1:**
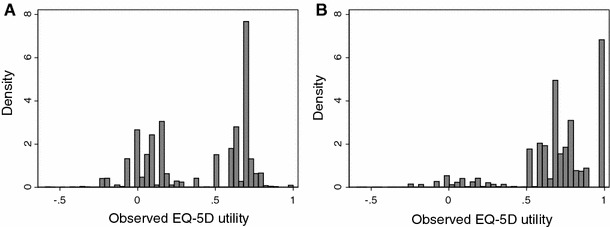
Distribution of **a** pre-operative and **b** post-operative EQ-5D utilities

Across both KAT and PROMs, patients had poor pre-operative HRQoL, with mean OKS of 18.6 (SD: 7.9; range: 0, 48; Table 1) and mean utility of 0.39 (SD: 0.32, range: −0.594, 1), which is lower than those reported for many forms of cancer or cardiovascular disease [27]. In particular, 87.5 % (92,124/105,235) of patients had problems with mobility, usual activities *and* pain pre-operatively. HRQoL improved substantially following knee replacement to a mean OKS of 33.8 (SD: 10.2; range: 0, 48) and mean utility of 0.70 (SD: 0.27; range: −0.594, 1). Like some previous mapping datasets [10], post-operative EQ-5D utilities followed a trimodal distribution (Fig. 1).

**Table 1:** Health-related quality-of-life scores for validation and estimation datasets

Question/score	KAT		PROMs		Combined estimation sample (*N* = 134,269)	Combined internal validation sample (*N* = 45,213)	External validation sample (EOC) (*N* = 10,002)
	Pre-op (*N* = 2,115)	Post-op (*N* = 15,301)	Pre-op (*N* = 103,120)	Post-op (*N* = 59,946)			
Proportion of patients at level 2 or level 3 on EQ-5D items							
Mobility							
L2:	96.2 %	57.2 %	93.4 %	52.6 %	76.9 %	77.2 %	64.2 %
L3:	0.5 %	0.1 %	0.4 %	0.1 %	0.3 %	0.3 %	0.43 %
Self-care							
L2:	29.7 %	24.3 %	32.8 %	21.0 %	28.1 %	28.3 %	26.7 %
L3:	0.2 %	0.5 %	1.0 %	0.5 %	0.8 %	0.8 %	1.2 %
Usual activities							
L2:	76.9 %	56.1 %	76.2 %	52.8 %	66.8 %	66.8 %	59.4 %
L3:	8.9 %	5.2 %	15.2 %	5.4 %	11.0 %	11.1 %	8.2 %
Pain and discomfort							
L2:	54.9 %	58.8 %	58.4 %	61.3 %	59.4 %	59.0 %	58.1 %
L3:	44.2 %	7.4 %	40.6 %	6.3 %	26.3 %	27.1 %	16.6 %
Anxiety/depression							
L2:	38.3 %	24.6 %	35.2 %	21.3 %	29.7 %	30.1 %	28.5 %
L3:	2.9 %	1.7 %	4.6 %	2.6 %	3.6 %	3.8 %	3.6 %
Mean (SD) HRQoL score							
EQ-5D utility	0.38 (0.31)	0.69 (0.27)	0.39 (0.32)	0.7 (0.27)	0.51 (0.34)	0.52 (0.34)	0.61 (0.32)
Total OKS	18.01 (7.57)	33.79 (10.43)	18.59 (7.95)	33.79 (10.2)	24.76 (11.67)	24.86 (11.68)	29.11 (11.70)
Usual level of pain	0.57 (0.67)	2.57 (1.25)	0.55 (0.66)	2.46 (1.18)	1.34 (1.32)	1.35 (1.32)	1.79 (1.42)
Trouble with washing and drying	2.76 (1.02)	3.35 (0.88)	2.79 (1.03)	3.43 (0.84)	3.04 (1.01)	3.05 (1.01)	3.13 (0.98)
Trouble with transport	1.93 (0.84)	2.74 (0.97)	2.06 (0.86)	2.88 (0.93)	2.38 (0.98)	2.38 (0.98)	2.52 (1.02)
Walking time before severe pain	1.79 (1.07)	3.1 (1.17)	1.99 (1.14)	3.17 (1.09)	2.46 (1.26)	2.47 (1.26)	2.79 (1.20)
Pain on standing up from sitting	1.59 (0.85)	3.01 (0.97)	1.64 (0.82)	2.94 (0.94)	2.17 (1.09)	2.18 (1.09)	2.6 (1.10)
Limping	0.81 (1.02)	2.98 (1.21)	0.88 (0.99)	2.88 (1.2)	1.7 (1.47)	1.71 (1.47)	2.29 (1.47)
Difficulty kneeling	0.70 (0.87)	1.23 (1.31)	0.79 (0.89)	1.4 (1.33)	1.02 (1.13)	1.02 (1.13)	1.40 (1.24)
Pain at night	1.34 (1.22)	2.94 (1.23)	1.26 (1.19)	2.64 (1.31)	1.85 (1.42)	1.86 (1.42)	2.33 (1.40)
Pain interferes with work	1.54 (0.88)	2.99 (1.05)	1.39 (0.88)	2.87 (1.07)	2 (1.21)	2.01 (1.21)	2.41 (1.23)
Sense of knee instability	1.89 (1.23)	3.43 (0.9)	1.85 (1.18)	3.37 (0.92)	2.48 (1.32)	2.48 (1.32)	2.86 (1.26)
Can do household shopping alone	1.52 (1.19)	2.82 (1.34)	1.67 (1.23)	2.89 (1.32)	2.16 (1.40)	2.17 (1.40)	2.53 (1.40)
Trouble walking down stairs	1.57 (0.91)	2.64 (1.16)	1.71 (0.92)	2.85 (1.08)	2.15 (1.13)	2.16 (1.13)	2.48 (1.11)

Patient observations with missing data on one or more questions in EQ-5D or OKS were omitted

*HRQoL* health-related quality of life, *KAT* Knee Arthroplasty Trial, *L2* Level 2 on EQ-5D, *L3* Level 3 on EQ-5D, *OKS* Oxford Knee Score, *PROMs* Patient Reported Outcome Measures [dataset], *SD* standard deviation, *EOC* Elective Orthopaedics Centre [dataset]

Total OKS was highly correlated with EQ-5D utility (*R* ^2^ = 0.61; *p* < 0.001). All OKS items showed significant Spearman’s rank correlations with all EQ-5D domains (*p* < 0.0001), and all OKS and EQ-5D questions loaded strongly onto a single component explaining 40 % of the variance in pre-operative scores and 54 % post-operatively. Plotting mean utility against OKS question ranking suggested that response levels were approximately linear with respect to utility for all OKS items other than pain (which showed a larger drop in utility between levels 0 and 1 than between other levels) and washing/drying, walking, shopping and going downstairs (which showed a much smaller drop between levels 0 and 1).

### Comparison of mapping model specifications

Eight mapping functions were evaluated using the combined estimation dataset (Table 2), which comprised 134,269 observations of 81,213 patients drawn from both the KAT and PROMs datasets.

**Table 2:** Comparison of performance across models investigated using combined dataset

Functional form	Dependent variable(s)	MSE		MAE	
		Internal validation	Estimation sample	Internal validation	Estimation sample
Direct utility mapping models					
OLS	EQ-5D utility	0.0363	0.0362	0.1398	0.1399
GLM (gamma family; identity link)	1 − EQ-5D utility	0.0397	0.0393	0.1467	0.1466
GLM (Gaussian family; log link)	1 − EQ-5D utility	0.0370	0.0368	0.1415	0.1415
Fractional logit	(EQ-5D utility + 0.594)/1.594	0.0367	0.0365	0.1403	0.1403
2-part models:		0.0360	0.0359	0.1384	0.1384
Part 1: logistic regression predicting perfect health^a^					
Part 2: OLS predicting utility given imperfect health					
3-part models:		0.0358	0.0357	0.1338	0.1341
Part 1: mlogit on perfect health, N3 or neither^b^					
Part 2: OLS on utility for pts with neither N3 nor perfect health					
Part 3: OLS on utility for N3 pts					
Response mapping models					
ologit response mapping	EQ-5D responses for each domain in turn	0.0359	0.0358	0.1361	0.1363
mlogit response mapping	EQ-5D responses for each domain in turn	0.0356	0.0354	0.1341	0.1343

*GLM* generalized linear model, *MAE* mean absolute error, *MSE* mean squared error, *OKS* Oxford Knee Score, *OLS* ordinary least squares

^a^Problems with perfect prediction arose when all response levels for washing/drying question were included; results are shown for a model merging levels 0 and 1 for this question

^b^Problems with non-symmetric or highly singular variance matrix arose when all response levels for washing/drying and work questions were included; results are shown for a model merging levels 0 and 1 for these questions

Across all functional forms, models using dummies indicating responses to OKS questions as explanatory variables produced better predictions than those using question or total scores (data not shown). However, all models using OKS responses as explanatory variables showed some logical inconsistencies in coefficient values that contradicted the implicit ordering whereby OKS response level 4 is unambiguously best, followed by level 3, 2, 1 and then 0.

Based on MSE, the primary measure of prediction accuracy, a response mapping algorithm using mlogit gave best predictions (MSE: 0.0356; Table 2), followed by the three-part model (MSE: 0.0358). However, the three-part model had lower MAE than mlogit (0.1338 vs 0.1341). The ologit response mapping (MSE: 0.0359), two-part model (MSE: 0.0360) and OLS (MSE: 0.0363) also performed reasonably well. However, fractional logit and GLM models gave relatively poor predictions (MSE: 0.0367–0.0397) and systematically underestimated utilities by an average of 0.00063–0.0025. The mlogit model also overestimated utilities for those with utility <0.5 by less than any other model (mean residual: 0.160, vs 0.162–0.170) but underestimated utilities for patients with utility ≥0.5 by a larger amount than any model other than ologit or GLM with gamma link (mean residual: −0.078, vs −0.075 to −0.076).

### Impact of dataset on results

**Fig. 2:**
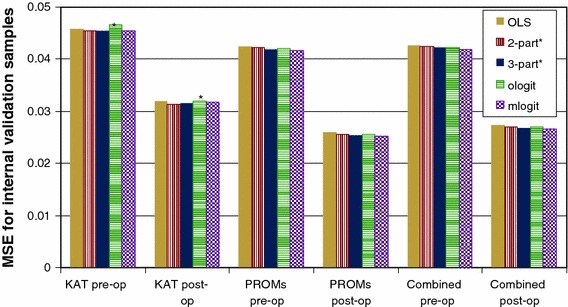
Mean squared error (MSE) for models fitted on each dataset. Models were tested in their respective internal validation samples: for example, the performance of models estimated using KAT was tested on 25 % of the KAT sample. *Levels 0/1 (or levels 3/4) were merged for one or more OKS item in logistic regression models as problems with perfect prediction or non-symmetric/highly singular variance matrices arose with the first-part model when all response levels were included

The relative performance of different model specifications differed between datasets (Fig. 2). When models were estimated using KAT data, a two-part model performed best in the KAT internal validation sample (MSE = 0.0331). However, mlogit performed best (MSE = 0.0356) among the models estimated on PROMs. Models estimated using the PROMs dataset gave more accurate predictions than those fitted on KAT for both pre-operative and post-operative observations. Models fitted on the PROMs or combined datasets also had up to 20 % fewer OKS items with counter-intuitive signs or rankings and converged more easily than those estimated using KAT.

We then fitted the best five models to the combined dataset with a full set of interaction terms capturing the effect of dataset on coefficient values. This suggested that 15–27 % of coefficients differed significantly between datasets (*p* < 0.05). Notably, all coefficients for the work OKS item were significantly higher and those for washing/drying were significantly lower for KAT than PROMs unless questionnaires more than 6 months post-operation were excluded, probably due to the effect of ageing and retirement during the longer KAT follow-up.

However, cross-validation prediction accuracy was good, with a two-part model fitted using KAT data having an MSE of 0.0361 in the PROMs validation sample and the mlogit PROMs model having an MSE of 0.0338 in KAT. The predictions from models estimated on the two datasets were also very similar: the mean absolute difference in predictions between the two-part model estimated on KAT data and the mlogit PROMs model was 0.031 (*p* < 0.0001), while the difference in predicted change from baseline at 3–6 months was 0.0042 (*p* = 0.003). Both models accurately predicted the mean utility in the other dataset: the mlogit PROMs model predicted the mean utility in KAT to be 0.665, vs an observed value of 0.654, while the two-part KAT model predicted the mean PROMs utility to be 0.496 vs an observed value of 0.503. Coefficient values and choice of model also differed between pre- and post-operative observations, although models of pre-operative utilities predicted post-operative utilities well (MSE: 0.0277) and vice versa (MSE: 0.0446).

### Performance of best model

Although the relationship between OKS and EQ-5D differed significantly between datasets and following knee replacement, the magnitude of such differences was very small. Furthermore, mapping models fitted on a heterogeneous population including both baseline and post-treatment observations and those from trials and routine clinical practice are likely to be useful for most practical applications. The mlogit response mapping model fitted on the combined estimation dataset was therefore selected as the best model since it gave the lowest MSE overall for both pre- and post-operative observations.

Adding patient sex into this model reduced MSE by only 0.0000073, to 0.03554. The final model (Table 3; Appendix 1: Electronic supplementary material) therefore included only OKS responses and was fitted on the entire KAT and PROMs dataset (including the internal validation sample).

**Table 3:** Regression coefficients for the best model: response mapping model of OKS responses fitted using multinomial logistic regression

OKS question and response level	Mean (SE) coefficient for each EQ-5D domain									
	Mobility		Self-care		Usual activities		Pain and discomfort		Anxiety and depression	
	L2 versus L1	L3 versus L1	L2 versus L1	L3 versus L1	L2 versus L1	L3 versus L1	L2 versus L1	L3 versus L1	L2 versus L1	L3 versus L1
Usual level of pain										
0	0.306 (0.053)*	−0.403 (0.475)	−1.654 (0.057)*	−1.95 (0.243)*	−0.432 (0.049)*	−0.754 (0.103)*	2.541 (0.087)*	3.729 (0.111)*	−0.11 (0.046)*	−0.41 (0.156)*
1	0.239 (0.04)*	0.286 (0.473)	−1.207 (0.055)*	−1.212 (0.234)*	−0.16 (0.039)*	−0.394 (0.097)*	3.12 (0.059)*	2.04 (0.092)*	−0.012 (0.044)	−0.407 (0.154)*
2	0.071 (0.036)	0.002 (0.502)	−0.661 (0.053)*	−0.473 (0.227)*	−0.03 (0.036)	−0.09 (0.095)	2.236 (0.04)*	0.898 (0.089)*	0.044 (0.044)	−0.098 (0.155)
3	−0.056 (0.031)	0.484 (0.443)	−0.278 (0.048)*	−0.036 (0.197)	−0.061 (0.03)*	−0.151 (0.088)	1.174 (0.029)*	0.333 (0.08)*	0.011 (0.04)	−0.272 (0.151)
Trouble with washing and drying										
0	0.581 (0.329)	2.107 (0.403)*	4.228 (0.148)*	6.06 (0.195)*	0.654 (0.326)*	1.621 (0.33)*	−0.084 (0.337)	−0.186 (0.339)	0.651 (0.078)*	1.089 (0.106)*
1	0.345 (0.094)*	1.195 (0.236)*	3.855 (0.037)*	3.578 (0.136)*	0.891 (0.100)*	1.339 (0.104)*	−0.305 (0.149)*	−0.251 (0.151)	0.726 (0.027)*	1.122 (0.056)*
2	0.378 (0.037)*	0.361 (0.217)	2.768 (0.024)*	1.386 (0.13)*	0.716 (0.035)*	0.861 (0.044)*	0.021 (0.061)	−0.035 (0.065)	0.513 (0.017)*	0.494 (0.048)*
3	0.227 (0.022)*	0.421 (0.223)	1.428 (0.023)*	0.688 (0.127)*	0.411 (0.021)*	0.446 (0.035)*	0.001 (0.032)	−0.038 (0.04)	0.223 (0.016)*	0.059 (0.051)
Trouble with transport										
0	−0.13 (0.239)	0.821 (0.479)	0.47 (0.105)*	0.551 (0.247)*	0.436 (0.283)	0.836 (0.294)*	−0.702 (0.246)*	−1.059 (0.252)*	0.336 (0.077)*	0.534 (0.133)*
1	0.22 (0.059)*	0.161 (0.409)	0.4 (0.044)*	0.031 (0.215)	0.523 (0.054)*	0.488 (0.075)*	−0.433 (0.092)*	−0.301 (0.101)*	0.356 (0.032)*	0.455 (0.095)*
2	0.211 (0.029)*	−0.013 (0.401)	0.244 (0.04)*	−0.227 (0.201)	0.467 (0.027)*	0.33 (0.056)*	0.049 (0.04)	−0.099 (0.056)	0.239 (0.027)*	0.164 (0.091)
3	0.097 (0.024)*	−0.356 (0.421)	0.102 (0.039)*	−0.442 (0.199)*	0.277 (0.022)*	0.141 (0.056)*	0.033 (0.028)	−0.069 (0.049)	0.141 (0.026)*	−0.015 (0.093)
Walking time before severe pain										
0	0.355 (0.041)*	1.307 (0.309)*	0.406 (0.034)*	0.529 (0.167)*	−0.045 (0.042)	0.332 (0.061)*	−0.267 (0.061)*	0.342 (0.071)*	0.132 (0.028)*	0.369 (0.075)*
1	0.863 (0.056)*	1.121 (0.317)*	0.484 (0.033)*	0.628 (0.164)*	0.084 (0.05)	0.552 (0.066)*	0.018 (0.084)	0.193 (0.091)*	0.184 (0.027)*	0.358 (0.074)*
2	0.991 (0.032)*	0.912 (0.316)*	0.331 (0.029)*	0.139 (0.164)	0.25 (0.029)*	0.448 (0.052)*	0.277 (0.053)*	0.429 (0.062)*	0.132 (0.023)*	0.257 (0.07)*
3	0.602 (0.022)*	−0.191 (0.391)	0.089 (0.028)*	−0.074 (0.168)	0.154 (0.022)*	0.128 (0.05)*	0.286 (0.032)*	0.146 (0.048)*	0.076 (0.021)*	0.082 (0.073)
Pain on standing up from sitting										
0	0.115 (0.161)	0.778 (0.522)	−0.086 (0.066)	0.277 (0.265)	−0.058 (0.138)	−0.463 (0.159)*	−0.314 (0.233)	0.7 (0.239)*	−0.028 (0.054)	0.209 (0.147)
1	−0.1 (0.049)*	−0.242 (0.487)	−0.149 (0.049)*	−0.005 (0.249)	−0.086 (0.044)	−0.569 (0.085)*	0.761 (0.09)*	1.085 (0.108)*	−0.118 (0.039)*	−0.24 (0.139)
2	−0.094 (0.036)*	−0.542 (0.49)	−0.088 (0.046)	0.193 (0.237)	0.002 (0.034)	−0.388 (0.079)*	0.861 (0.051)*	0.534 (0.079)*	−0.073 (0.037)*	−0.223 (0.136)
3	−0.06 (0.028)*	−0.276 (0.416)	−0.056 (0.042)	0.145 (0.21)	0.03 (0.026)	−0.238 (0.072)*	0.508 (0.027)*	0.082 (0.065)	−0.064 (0.033)	−0.192 (0.129)
Limping										
0	1.63 (0.046)*	1.586 (0.376)*	−0.369 (0.045)*	−0.852 (0.19)*	0.479 (0.042)*	0.698 (0.079)*	0.602 (0.073)*	0.700 (0.092)*	−0.067 (0.036)	−0.126 (0.116)
1	1.284 (0.037)*	1.452 (0.375)*	−0.357 (0.043)*	−0.625 (0.19)*	0.277 (0.036)*	0.309 (0.077)*	0.693 (0.063)*	0.509 (0.084)*	−0.029 (0.035)	−0.079 (0.115)
2	1.067 (0.039)*	0.796 (0.475)	−0.183 (0.045)*	−0.657 (0.213)*	0.246 (0.037)*	0.218 (0.081)*	0.544 (0.062)*	0.214 (0.086)*	0.096 (0.036)*	0.049 (0.121)
3	0.502 (0.025)*	0.505 (0.391)	−0.053 (0.038)	−0.372 (0.181)*	0.177 (0.025)*	0.084 (0.07)	0.233 (0.027)*	−0.139 (0.063)*	0.072 (0.031)*	0.011 (0.109)
Difficulty kneeling										
0	0.464 (0.05)*	−0.92 (0.931)	0.678 (0.105)*	−0.123 (0.467)	0.812 (0.049)*	0.375 (0.168)*	0.456 (0.052)*	0.459 (0.128)*	0.315 (0.068)*	0.882 (0.315)*
1	0.293 (0.051)*	−0.686 (0.935)	0.507 (0.106)*	−0.072 (0.469)	0.704 (0.05)*	0.224 (0.168)	0.362 (0.054)*	0.383 (0.129)*	0.236 (0.068)*	0.795 (0.316)*
2	0.173 (0.049)*	−0.482 (0.961)	0.313 (0.105)*	0.043 (0.475)	0.525 (0.048)*	−0.03 (0.168)	0.243 (0.049)*	0.025 (0.128)	0.195 (0.067)*	0.63 (0.317)*
3	0.077 (0.048)	0.05 (1.019)	0.245 (0.104)*	0.133 (0.483)	0.301 (0.047)*	−0.242 (0.174)	0.082 (0.047)	−0.141 (0.13)	0.106 (0.067)	0.535 (0.317)
Pain at night										
0	−0.369 (0.042)*	−0.519 (0.24)*	0.093 (0.034)*	−0.035 (0.147)	−0.172 (0.039)*	−0.536 (0.058)*	1.296 (0.092)*	2.024 (0.098)*	0.172 (0.027)*	0.417 (0.077)*
1	−0.239 (0.036)*	−0.449 (0.253)	0.082 (0.033)*	0.015 (0.15)	−0.182 (0.033)*	−0.41 (0.054)*	1.225 (0.068)*	1.474 (0.077)*	0.132 (0.026)*	0.233 (0.079)*
2	−0.093 (0.026)*	−0.276 (0.251)	0.064 (0.03)*	0.023 (0.14)	−0.107 (0.026)*	−0.231 (0.049)*	0.882 (0.032)*	0.864 (0.049)*	0.119 (0.024)*	0.096 (0.077)
3	−0.084 (0.028)*	−0.173 (0.36)	0.062 (0.035)	−0.182 (0.187)	−0.036 (0.027)	−0.176 (0.06)*	0.368 (0.029)*	0.258 (0.06)*	0.098 (0.028)*	0.103 (0.096)
Pain interferes with work										
0	0.993 (0.091)*	2.411 (0.54)*	0.333 (0.057)*	0.842 (0.28)*	2.493 (0.088)*	4.432 (0.127)*	0.287 (0.148)	0.855 (0.161)*	0.748 (0.046)*	1.186 (0.16)*
1	0.917 (0.051)*	1.396 (0.526)*	0.103 (0.053)	0.051 (0.276)	2.539 (0.048)*	3.371 (0.101)*	0.654 (0.088)*	0.917 (0.108)*	0.555 (0.041)*	0.664 (0.157)*
2	0.638 (0.034)*	0.938 (0.512)	−0.059 (0.05)	0.185 (0.264)	1.684 (0.033)*	1.589 (0.093)*	0.749 (0.049)*	0.444 (0.081)*	0.345 (0.038)*	0.241 (0.153)
3	0.348 (0.026)*	1.013 (0.476)*	−0.011 (0.044)	0.02 (0.232)	1.013 (0.025)*	0.488 (0.087)*	0.384 (0.028)*	−0.005 (0.07)	0.204 (0.034)*	0.154 (0.143)
Sense of knee instability										
0	0.198 (0.078)*	0.257 (0.299)	−0.03 (0.036)	0.405 (0.167)*	0.036 (0.069)	0.165 (0.081)*	0.063 (0.139)	0.386 (0.143)*	0.273 (0.03)*	0.735 (0.075)*
1	0.26 (0.047)*	0.136 (0.283)	−0.118 (0.03)*	0.309 (0.164)	−0.066 (0.041)	−0.09 (0.057)	0.078 (0.091)	0.21 (0.097)*	0.248 (0.025)*	0.387 (0.072)*
2	0.268 (0.037)*	−0.142 (0.315)	−0.128 (0.029)*	0.371 (0.165)*	0.071 (0.034)*	−0.026 (0.054)	0.277 (0.072)*	0.225 (0.079)*	0.223 (0.024)*	0.225 (0.074)*
3	0.134 (0.022)*	−0.537 (0.306)	−0.112 (0.026)*	0.135 (0.15)	0.05 (0.022)*	0.024 (0.045)	0.169 (0.029)*	0.053 (0.045)	0.174 (0.021)*	0.155 (0.07)*
Can do household shopping alone										
0	1.837 (0.052)*	3.564 (0.455)*	2.005 (0.045)*	3.008 (0.292)*	1.553 (0.048)*	2.975 (0.084)*	0.54 (0.07)*	1.385 (0.085)*	0.952 (0.033)*	1.747 (0.124)*
1	1.791 (0.055)*	2.542 (0.481)*	1.727 (0.045)*	1.896 (0.302)*	1.464 (0.049)*	2.359 (0.086)*	0.482 (0.083)*	1.319 (0.096)*	0.862 (0.032)*	1.529 (0.124)*
2	1.395 (0.032)*	2.661 (0.456)*	1.373 (0.043)*	1.631 (0.298)*	1.148 (0.03)*	1.654 (0.075)*	0.427 (0.051)*	0.901 (0.069)*	0.607 (0.029)*	1.068 (0.121)*
3	0.896 (0.022)*	1.891 (0.478)*	0.925 (0.041)*	1.084 (0.282)*	0.721 (0.022)*	0.952 (0.071)*	0.293 (0.031)*	0.662 (0.056)*	0.37 (0.027)*	0.758 (0.118)*
Trouble walking down stairs										
0	1.300 (0.087)*	3.614 (0.468)*	1.048 (0.057)*	2.242 (0.309)*	0.751 (0.077)*	1.702 (0.116)*	0.347 (0.107)*	0.964 (0.128)*	0.351 (0.044)*	0.316 (0.139)*
1	1.160 (0.049)*	2.23 (0.463)*	0.855 (0.05)*	1.194 (0.308)*	0.798 (0.043)*	1.341 (0.096)*	0.593 (0.074)*	1.346 (0.097)*	0.342 (0.037)*	0.287 (0.133)*
2	0.921 (0.03)*	1.524 (0.449)*	0.64 (0.047)*	0.984 (0.301)*	0.683 (0.029)*	1.088 (0.089)*	0.554 (0.042)*	0.844 (0.075)*	0.276 (0.034)*	0.179 (0.131)
3	0.602 (0.024)*	0.174 (0.543)	0.412 (0.045)*	0.761 (0.287)*	0.447 (0.023)*	0.814 (0.085)*	0.314 (0.027)*	0.509 (0.066)*	0.181 (0.032)*	0.018 (0.126)
Constant	−2.444 (0.045)*	−10.015 (1.406)*	−4.099 (0.1)*	−7.455 (0.483)*	−2.441 (0.045)*	−5.325 (0.157)*	−2.453 (0.045)*	−4.726 (0.12)*	−2.976 (0.062)*	−6.275 (0.291)*

*L1* level 1 on the EQ-5D domain in question (no problems), *L2* level 2 on the EQ-5D domain in question (some problems), *L3* level 3 on the EQ-5D domain in question (severe problems), *OKS* Oxford Knee Score, *SE* standard error

* *p* < 0.05

Excel and Stata code (oks2eq) to estimate predictions from this model are available at http://www.herc.ox.ac.uk/downloads

**Fig. 3:**
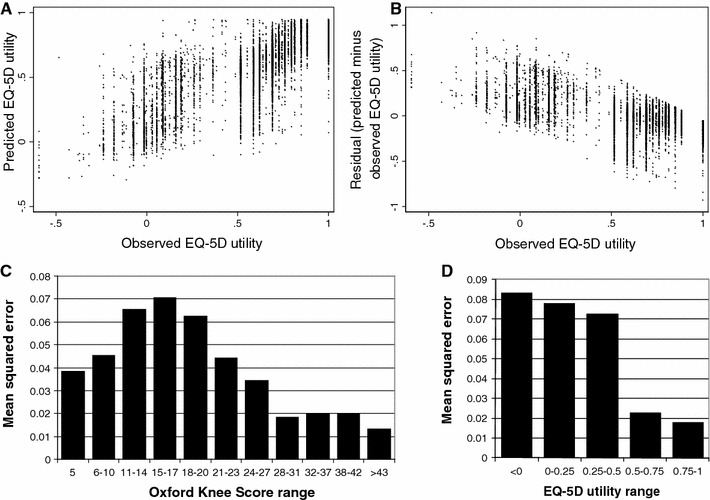
Performance of the best model in the external validation sample. **a** Scatter plot showing correlation between observed and predicted EQ-5D utility. **b** Scatter plot showing correlation between residual (predicted minus observed EQ-5D utility) and observed EQ-5D utility. **c** Mean squared error by OKS. **d** Mean squared error by observed EQ-5D

The final model accurately predicted EQ-5D utility in the combined KAT/PROMs sample (MSE: 0.0355; MAE: 0.134) and the external EOC sample (MSE: 0.0330; MAE: 0.129). Within EOC, 18 % of predictions were within 0.05 and 42 % within 0.10 of the observed utility value; predicted and observed utilities were strongly correlated (*R* ^2^: 0.69; Fig. 3a). The predicted proportions of patients with different response levels on each domain were very similar, but were significantly different from the observed proportions (*p* < 0.0001, based on chi-squared test in Microsoft Excel 2003): for example, the model predicted that 26 % of EOC questionnaires indicated some anxiety and depression, compared with the 28.5 % (2,848/10,002) observed. The model also accurately predicted mean utility (observed: 0.607; predicted: 0.597) in EOC. Like most mapping models [10], ceiling and floor effects produced heteroskedastic residuals, causing our model to slightly underestimate utilities for patients with high EQ-5D utility and overestimate utility for patients with low utility (Fig. 3b). Predicted utilities also had a smaller range (−0.29 to 0.95 vs −0.594 to 1) and standard deviation (0.26 vs 0.32) than observed values.

For all models, prediction accuracy was better for post-operative observations (Fig. 2) and observations with high utility (Fig. 3d) and markedly worse for patients with OKS between 11 and 20 than for those with better or worse knee function (Fig. 3c). Within the KAT baseline sample, predictions were also less accurate (MSE: 0.043) for those with poor pre-operative general health (ASA grade 3–4) and those with arthritis in other joints. The final model also accurately predicted change in utility following knee replacement (MSE: 0.0656; MAE: 0.192).

## Discussion

We have developed a mapping algorithm that accurately predicts EQ-5D utility based on OKS responses; model performance was similar to previous mapping models, which have obtained MAEs between 0.0011 and 0.19 [8]. The mapping model shown in Table 3 can be used to predict responses to the EQ-5D questionnaire and EQ-5D utilities in situations where only OKS has been administered. In particular, this will facilitate cost-utility analyses of the numerous trials and registries that used OKS but no utility measure. Excel and Stata code developed to estimate predictions are available at http://www.herc.ox.ac.uk/downloads, and methods to estimate standard errors around predictions from the variance–covariance matrices of response mapping models (Appendix 1) are under development. The models described require patient-level data on OKS responses, although a simpler model for secondary data is available at http://www.herc.ox.ac.uk/downloads. However, mapping is no substitute for including a utility measure in future studies and does not overcome the limitations of either instrument [10].

In addition to producing more accurate predictions in this study, response mapping models naturally deal with non-Gaussian utility distributions and mirror the way utilities are calculated. Furthermore, while direct mapping models must be developed for specific tariffs, response mapping algorithms can be applied to any three-level EQ-5D tariff available now or in the future [11]. Although prediction accuracy varied with tariff, our algorithm gave accurate predictions of utilities in the external validation sample using the EQ-5D tariffs for Spain, Germany, Netherlands, Denmark, Japan, Zimbabwe and USA (MSE ≤ 0.055). Response mapping also gives richer insights into the relationship between the two instruments, for instance predicting the proportion of patients with different response levels on each domain. However, such models appear to perform much better when estimated on very large datasets. The three-part model specification we developed to deal with the N3 term in the UK EQ-5D tariff also performed very well; this specification may be particularly useful for other mapping applications where severe problems on EQ-5D domains are common.

Our dataset is (to our knowledge) the largest sample used for mapping analyses to date and covers the full range of EQ-5D and OKS scores. In particular, our large sample size appears to have overcome previously cited difficulties with mapping between Oxford Hip Score and EQ-5D, such as lack of overlap between pre- and post-operative scores and poor prediction of anxiety and depression [28]. Although the model performed well overall, predictions were less accurate for patients with OKS between 11 and 20, which appears to be due to uncertainty about which 54.3 % of such patients have severe problems on ≥1 domain, since MSE did not vary markedly with OKS when observed and predicted utilities were recalculated without the N3 term. The N3 term may also explain the general finding of higher accuracy for healthier patients [8]. However, the performance of our mapping algorithm in populations dissimilar to ours (e.g. patients with early arthritis) or for studies using non-English language questionnaires is unknown.

Although OKS includes questions directly relating to mobility, self-care, usual activities and pain, no OKS questions directly ask about psychological symptoms or strongly predict responses to the EQ-5D anxiety/depression question (mlogit pseudo-*R* ^2^: 0.14 for anxiety/depression, vs 0.36–0.55 for other domains). Nonetheless, we found that OKS predicts anxiety/depression responses reasonably accurately, probably as pain and poor knee function explain much of the anxiety/depression observed in this population. Nonetheless, any mapping algorithm between OKS and EQ-5D is likely to perform poorly in subgroups of patients who have psychological conditions that unrelated to their knee problems. Our mapping algorithm was also less accurate in patients with comorbidities or arthritis in other joints, probably due to OKS’ focus on knee problems.

Models using dummies indicating OKS response level as the explanatory variable gave better predictions than those modelling total or question scores. This demonstrates the advantages of modelling response levels for each question whenever the estimation dataset is large enough to estimate coefficients reliably. Regression analyses also indicate that some items (e.g. pain or impact on work) have more effect on utility than others (Table 3). OKS total score was nonetheless a strong predictor of EQ-5D, suggesting that the OKS scoring system (which assigns equal weight to all questions and assumes levels are equally spaced given the wording of questions and response levels) is a good measure of HRQoL.

However, coefficients for some OKS response levels had counter-intuitive signs or rankings (Table 3): for example, the coefficients showing the effect of being unable to walk at all without severe pain (0.35) or being able to walk only around the house (0.86) on having level 2 mobility were lower than the coefficient for walking 5–15 min (0.99). Such inconsistencies were less common in mapping models fitted on the larger PROMs dataset than on KAT, although 57 % of OKS items were inconsistent in the final model (Table 3). Similar inconsistencies have been observed previously [8, 11, 29]. These inconsistencies could cause the mapping algorithm to predict that a patient’s utility had fallen when their OKS profile was unambiguously improved. In principle, items could be omitted or levels merged to give a fully consistent mapping algorithm with higher face validity: particularly as the specific inconsistencies observed appeared to vary between datasets, suggesting that many such inconsistencies occurred by chance. However, we feel that it is more appropriate to use the mapping model giving highest prediction accuracy in the validation sample regardless of inconsistencies, rather than applying ad hoc methods that could give many different “consistent” algorithms. Furthermore, we found that omitting/merging OKS levels reduced prediction accuracy, suggesting that inconsistencies may reflect patients’ interpretation of the questions or genuine opposition between items.

Model choice and coefficient values were sensitive to the dataset used to estimate mapping models. However, while the predictions and coefficients differed significantly between datasets, such differences are unlikely to be large enough to affect the results of an economic evaluation: particularly as differences in change from baseline were smaller than those for absolute values. Longer post-operative follow-up in KAT may explain many of these differences, although differences could also arise from slight differences in methods of questionnaire administration/wording or secular trends between operation dates (1999–2003 for KAT and 2009–2010 for PROMs). Other explanations, such as differing patient characteristics or questionnaire translations, are unlikely in this case as cohorts were similar and all questionnaires were completed in English. Although the relationship between OKS and EQ-5D appears to differ slightly between pre- and post-operative observations, using different mapping algorithms for different timepoints could bias cost-effectiveness estimates. Furthermore, adding observations from trial data with long follow-up (KAT) to those from routine data (PROMs) is likely to increase the range of applications to which mapping algorithms can be applied.

Nonetheless, differences between datasets highlight the importance of external validation. Selecting models based on performance in an internal validation dataset not used in model estimation helps prevent over-fitting, while external validation provides a more rigorous test of predictive accuracy by assessing performance in a separate, independently collected dataset that was not used for model estimation or selection [10, 30]. Our model gave accurate predictions in both internal and external validation datasets, demonstrating that it is likely to perform well in other comparable populations.

## Electronic supplementary material

Below is the link to the electronic supplementary material.

Supplementary material 1 (XLSX 3112 kb)

## Abbreviations

EOC: Elective Orthopaedics Centre [dataset]
GLM: Generalized linear model
HRQoL: Health-related quality of life
KAT: Knee Arthroplasty Trial
MAE: Mean absolute error
MSE: Mean squared error
OLS: Ordinary least squares
OKS: Oxford Knee Score
PROMs: Patient Reported Outcome Measures [initiative]
QALY: Quality-adjusted life-year
